# Associations Between Serum Uric Acid Concentrations and Cardiometabolic Risk and Renal Injury in Obese and Overweight Children

**DOI:** 10.4274/jcrpe.galenos.2018.2019.0241

**Published:** 2019-09-03

**Authors:** Deniz Özalp Kızılay, Semra Şen, Betül Ersoy

**Affiliations:** 1 Çiğli State Training Hospital, Clinic of Pediatrics, Division of Pediatric Endocrinology, İzmir, Turkey; 2 Manisa Celal Bayar University Faculty of Medicine, Department of Pediatrics, Division of Pediatric Infectious Disease, Manisa, Turkey; 3 Manisa Celal Bayar University Faculty of Medicine, Department of Pediatrics, Division of Pediatric Endocrinology and Metabolism, Manisa, Turkey

**Keywords:** Serum uric acid concentration, obesity, metabolic syndrome, insulin resistance, renal injury, cardiovascular risk, child

## Abstract

**Objective:**

The aim of this study was to assess the association between serum uric acid concentration (SUAC) and the parameters of the metabolic syndrome (MetS) and insulin resistance (IR). The secondary aim was to evaluate whether hyperuricemia is associated with renal injury and cardiovascular risk in obese (OB) and overweight (OW) children.

**Methods:**

The subjects of this study consisted of OB/OW children and adolescents (ages: 8-18 years). Sex and age specific serum uric acid (SUA) olarak değiştirilecek percentiles were used and a SUA >75^th^ percentile was accepted as hyperuricemia. Anthropometric data, blood pressure (BP) measurements and biochemical parameters, including fasting blood glucose, insulin, total cholesterol, high-density lipoprotein cholesterol (HDL-c), low-density lipoprotein cholesterol, triglycerides (TG), aspartate aminotransferase, alanine aminotransferase, homeostatic model assessments of IR (HOMA-IR) and SUAC were recorded. Oral glucose tolerance tests (OGTT) were performed in all patients. MetS was defined according to the International Diabetes Federation criteria. Total cholesterol/HDL-c ratio >4 and TG/HDL-c ratio >2.2 were used as the atherogenic index (AI) indicating cardiovascular risk. Urinary albumin excretion in a 24-hour and also in a first-morning urine sample were measured. Renal injury was assessed by microalbuminuria according to the National Kidney Foundation criteria.

**Results:**

There were 128 participants; 52 (40%) had elevated (SUA >75^th^ percentile) and 76 had (60%) normal SUAC. The mean±SD age was 13.1±2.6 years and 87 (67.4%) were female. The mean±SD weight was 73±18.97 kg and mean±SD height was 155.4±12.11 cm. There was no statistical difference between the groups with and without hyperuricemia in terms of age, sex, puberty stage and degree of obesity. Increased SUAC were significantly associated with higher waist-to-hip ratio (WHR), fasting insulin levels and insulin at 30 and 60 minutes during OGTT, HOMA-IR, lower HDL-c and presence of hypertriglyceridemia as well as with decreased HDL-c, increased AI, presence of IR and MetS. BP and microalbuminuria were not associated with SUAC. SUAC showed significant positive correlations with waist circumference, WHR, post-challenge glucose level at 60 minutes, with fasting insulin, post-challenge insulin levels at 30, 60, 90 and 120 minutes and also with HOMA-IR, total cholesterol/HDL-c ratio, TG/HDL-c ratio and a number of other criteria related to MetS. Also, an inverse correlation with HDL-c was noted.

**Conclusion:**

In OB/OW children frequency of MetS, IR and dislipidemia increases with increased SUAC, a finding independent of age, puberty, gender and body mass index. Patients meeting all of the MetS criteria had the highest SUAC. These results demonstrate that the association between UA and metabolic and cardiovascular risk factors can be detected early in childhood. Thus, we recommend monitoring SUAC in OB children and we believe that prevention of SUAC elevation in early life has a potential protective effect on metabolic impairment and subsequent comorbidities.

**What is already known on this topic?**

In obese (OB) adults and adolescents, higher uric acid (UA) concentrations are associated with the risk factors characterizing the metabolic syndrome (MetS) and also with fasting insulin level and insulin resistance (IR). All these factors are predictive for both cardiovascular diseases and type 2 diabetes. Despite the knowledge that UA is associated with obesity-related comorbidities such as MetS, cardiovascular risk factors and kidney diseases, studies in overweight (OW) and OB children are rare and the results are still controversial.

**What this study adds?**

This study confirms associations of elevated serum UA with greater waist-to-hip ratio, lower HDL-cholesterol and hypertriglyceridemia, as well as with the presence of MetS and IR in OB and OW children. Moreover, the number of criteria related to MetS is significantly associated with the elevation of UA.

## Introduction

Uric acid (UA) is the end-product of purine metabolism, produced by the liver and excreted by the kidneys (1). Serum UA concentration (SUAC) increases progressively with body growth from early childhood until the ages of 15-17 years (2). Obese (OB) individuals have higher SUAC than in normal-weight peers. Hyperuricemia and obesity probably influence one another in many ways, depending on multiple mechanisms. Hyperuricemia may contribute to development of obesity by accelerating hepatic and peripheral lipogenesis (3). On the other hand, obesity may lead to serum UA (SUA) elevation due to several factors, such as OB subjects having reduced renal clearance of UA and obesity being associated with elevated activity of xanthine oxidase and increased production of UA by adipose tissue (4).

The increase in SUAC is an independent risk factor for lifestyle related diseases such as hypertension, renal diseases, cardiovascular diseases and also has a potential role in the development of the metabolic syndrome (MetS),  hyperinsulinemia and IR measured by the homeostatic model assessment of insulin resistance (HOMA-IR) (5). However, the relationship between obesity-related metabolic risk factors and SUAC in childhood is still controversial. While some studies report a strong association between these variables (6,7), others did not confirm an independent association (8,9).

In this study we aimed to investigate, whether increased SUAC is related to MetS risk factors, using standard methodology (10). We also aimed to evaluate the association between hyperuricemia with renal injury or cardiovascular risk factors in OB and overweight (OW) children.

## Methods

### Study Population

Children who visited the Pediatric Endocrinology Outpatient Clinic for general obesity screening were enrolled in the study. Ethics committee approval was obtained from Manisa Celal Bayar University (20.478.486). A total of 128 OB and OW children of ages 8 to 18, with a body mass index (BMI) greater than the 85^th^ percentile for age and gender according to the Center for Disease Control and Prevention (CDC-2000) data (11), were included in the study. The patients were divided into two groups according to their SUAC.

Children with type 1 or type 2 diabetes or whose obesity was related to a syndrome (Prader-Willi syndrome, Laurence-Moon Biedl syndrome, etc.) or to an endocrinologic condition such as Cushing’s syndrome or hypothyroidism were excluded. Subjects referred to our clinic for conditions related to obesity (e.g. alterations in bloodglucose levels, arterial hypertension, dyslipidemia, liver steatosis, hyperuricemia etc.), children who have received or are currently receiving treatments such as glucose or lipid-lowering drugs and/or anti-hypertensive medication, children with liver, kidney or other systemic diseases and family history of symptomatic hyperuricemia were also excluded from the study.

### Procedures

Physical examination and laboratory results of all subjects were recorded. All of the evaluations were conducted by specially trained clinical research staff.

### Anthropometric and Clinical Measurements

Height and weight were measured by a wall-mounted stadiometer for height and a calibrated scale for weight. The weight of each subject was measured with all clothing and shoes removed except undergarments. Waist circumference (WC) was measured with a non-stretchable tape to the nearest 0.1 cm midway between the lowest rib and the highest point of the iliac crest parallel to the floor, in a standing and relaxed position and during expiration. Hip circumference was measured at the widest portion of the  buttocks. Waist-to hip ratio (WHR) was calculated. Pubertal development stage was recorded according to Tanner classification. Blood pressure (BP) was measured with the right arm in the supine position after a five-minute rest, using a mercury sphygmomanometer with an appropriately sized cuff, and a stethoscope placed over the brachial artery pulse; three systolic and diastolic BP (SBP, DBP) measurements were taken two minutes apart and the mean of the two last values was used in data analyses.

### Laboratory Tests

Results of assessment of biochemical analytes including serum glucose, urea, creatinine, aspartate aminotransferase, alanine aminotransferase, total cholesterol, low-density lipoprotein cholesterol (LDL-c), high-density lipoprotein cholesterol (HDL-c), triglycerides (TG) and SUA were recorded. Each child underwent an oral glucose tolerance test (OGTT) following an overnight fasting of 12-14 hours. After subjects ingested a glucose solution containing 1.75 g/kg glucose (maximum 75 g), blood samples were obtained every 30 min for 120 min, for measurement of plasma glucose and insulin. In all subjects, the first-morning urine specimen was analyzed for albumin and creatinine. Urine was collected for 24 hours, and urinary albumin was measured. Samples showing pyuria and hematuria were excluded.

Total body obesity was estimated by BMI, central obesity measured by WHR or WC, atherogenic dyslipidemia by increased TG, decreased HDL-c and increased ratio of total cholesterol/HDL-c and TG/HDL-c. Presence of systolic and diastolic hypertension and hyperglycemia including fasting blood glucose level and/or abnormal glucose responses on OGTT, hyperinsulinemia and IR measured by the homeostatic model assessments of IR (HOMA-IR) were estimated in all subjects (10).

BMI was calculated by the standard formula (weight in kg divided by the square of height in metres). BMI standard deviation score (BMI SDS) and BMI percentiles were calculated using age and gender specific norms published by the CDC (11). Obesity was defined as BMI ≥95^th^ percentile and OW as BMI ≥85^th^ percentile for age and sex. The extent of obesity was quantified using Cole’s LMS method which stratifies obesity on the basis of a threshold BMI Z-score of 2.0 or more, namely, moderate obesity as a Z-score of 2.0-2.5, and severe obesity as a Z-score above 2.5 (12). WC percentiles were stratified according to sex and age, identifying abdominal obesity as a WC ≥90^th^ percentile as previously described (13). WHR was used as an index of fat distribution. A testicular volume of ≥4 mL in males, and breast development of Tanner stage 2 and over in females, were considered as findings of puberty (14).

IR was evaluated with the aid of HOMA-IR index using a standard formula: fasting insulin (µU/mL) x fasting glucose (mmol/L) divided by 22.5. IR criteria were HOMA-IR >2.5 for prepubertal children and HOMA-IR >4.0 for adolescents (15). Impaired fasting glucose was defined as a fasting plasma glucose level between 100 and 125 mg/dL without a history of diabetes mellitus (16). Impaired glucose tolerance was defined according to World Health Organization criteria, a condition in which fasting blood glucose levels in venous plasma drop to <140 mg/dL and the 120 minute post challenge blood glucose is between 140 and 200 mg/dL (16).

Hyperinsulinemia was defined as a fasting insulin ≥15 µU/mL, or  an insulin level during the OGTT test of ≥150 µU/mL and/or ≥75 µU/mL at 120 minutes following the start of the OGTT (17).

MetS was defined according to the International Diabetes Federation criteria (17). MetS can be diagnosed in children 10 to 16 years old when the following criteria are fulfilled: a WC ≥90^th^ percentile (sex and age specific), together with two or more risk factors. These risk factors are: 1) fasting blood glucose levels ≥100 mg/dL (5.6 mmol/L); 2) serum TG concentration ≥150 mg/dL (1.7 mmol/L) or treatment for elevated TG; 3) a low HDL-c <40 mg/dL (1.03 mmol/L) or treatment for low HDL-c; 4) either SBP ≥130 mmHg or DBP ≥85 mmHg, or treatment for hypertension, or a SBP level of at least 95^th^ percentile for sex, age and height (18).

For children 16 years and older, the adult criteria can be used (19). MetS can not be diagnosed in children younger than 10 years of age, but vigilance is recommended if the WC is ≥90^th^ percentile (20). Total cholesterol/HDL-c ratio is defined as the atherogenic index (AI), according to which a ratio of >4 (normal=2.5) is considered as a cardiovascular risk (20). The TG to HDL-ratio >2.2 was also considered as a marker of atherogenic risk (21). Hypertension was defined as a value above the 95^th^ percentile for age and height according to the National Health and Nutrition Examination Survey (22). Microalbuminuria in children and adolescents was defined as a urinary albumin excretion rate of 30-300 mg/24 hours and 3-30 mg/mmoL creatinine (30-300 mg/g creatinine) in a first-morning urine sample (23). Hyperuricemia was defined as SUA value ≥75^th^ percentile, adjusted for age and sex (24).

### Statistical Analysis

Continuous variables were expressed as mean±standard deviation (SD) and categorical variables as numbers and percentages. Normal distribution was tested using the Kolmogorov-Smirnov test. Between-group comparison for categorical variables was performed by using the χ^2^ test or Fisher’s exact tests. Student’s t-tests and Mann-Whitney U test were used for comparison of continuous variables.Correlations were investigated using Pearson’s correlation test. Statistical analyses were performed using the Statistical Package for Social Sciences 15.0 program (SPSS 15.0; IBM Inc., Chicago, Ill., USA). P values <0.05 were considered statistically significant.

## Results

In this study, 128 OB and OW children/adolescents were evaluated. Of these 52 (40%) had elevated SUA defined as SUAC ≥75^th^ percentile, adjusted for age and sex and 76 (60%) had normal SUAC. The mean age of the participants was 13.1±2.6 (range 8-18) years and 87 (67.4%) were female. Clinical and laboratory variables were compared in children with and without hyperuricemia and the results are presented in Table 1. The group with hyperuricemia was not statistically different from the group without hyperuricemia in terms of age, sex, puberty stage and degree of obesity. Subjects with hyperuricemia had higher WHR and lower HDL-c compared with those with normal SUAC. Moreover, subjects with hyperuricemia who showed higher insulin levels either at fasting or as responses to OGTT at 30 and 60 minutes and also tended to have higher IR values than those without hyperuricemia but this latter parameter did not reach significance.

Increased SUAC was significantly associated with the criteria related to MetS. Table 2 shows that elevated SUAC were significantly associated with hypertriglyceridemia, decreased HDL-c, increased AI, presence of IR and MetS.

Table 3 shows the results of the correlation analysis between the variables with SUAC in all subjects. SUAC showed a significant positive correlation with WC, WHR, post-challenge glucose level at 60 minutes, fasting insulin, post-challenge insulin levels at 30, 60, 90 and 120 minutes, HOMA-IR, total cholesterol to HDL-c ratio, TG to HDL-c ratio, criteria related to MetS and an inverse correlation with HDL-c.

## Discussion

Physiological UA concentrations have antioxidant and endothelial protective effects in the extracellular environment. However, increased SUAC has been reported to play a pro-oxidant role and might promote several harmful effects (25). A relationship between increased SUAC and obesity-related comorbidities such as MetS, IR, cardiovascular risk factors and kidney diseases has been reported in OB adults and children (26,27). However, the results of these studies are still controversial.

Epidemiological studies on large populations have shown that the prevalence of MetS shows a gradual increase with increased SUAC (28). Despite the apparent role of SUA in contributing to MetS related metabolic impairment, studies in OW/OB children are rare. In the study of Ford et al. (7) which included 1370 adolescents aged between 12-17 years, patients meeting all the MetS criteria were found to have the highest SUAC. In the STYJOBS/EDECTA cohort study of 299 OW/OB Japanese children aged between 8-18 years, SUA was shown as the best predictor of unhealthy obesity. Patients in the highest quartile of the SUAC were found to be heavier, with worse lipid and insulin metabolism. The authors suggested that hyperuricemia should be considered as a cardiometabolic risk factor in early childhood (29). Our study confirms that the presence of MetS and the criteria related to MetS are significantly associated with elevated SUAC in OB/OW children. A growing number of studies suggest that UA should be added to the list of criteria used to diagnose MetS (30,31). Thus SUA requires more attention in the evaluation of the metabolic risk profile of OB children and adolescents. The pattern of fat distribution, rather than BMI, is important for metabolic and cardiovascular diseases (32). Our results showed that increased SUAC was significantly associated with greater WHR and correlated with higher WC. The strong association found by us and others with WC confirms the strong link between UA and visceral adiposity (7,24). The association of UA with regional distribution of abdominal adipose tissue in children is poorly understood. Increased dietary fructose consumption leads to hepatic lipogenesis, thus contributing to increased visceral fat accumulation and ultimately worsening of IR (33). In addition, dietary fructose activates the fructokinase metabolic system and upregulates *de novo* purine nucleotide synthesis in hepatocytes, thereby causing an increase SUA production and hyperuricemia (34). High SUAC-associated dyslipidemia has been shown to be a result of low serum HDL-c levels, not increased LDL or VLDL levels (15). This study confirmed associations of elevated SUA with lower HDL-c and hypertriglyceridemia. Recent evidence suggests that UA induces vascular inflammation and artery damage, leading to increased risk of atherosclerosis. Findings of the present study confirmed an association between SUA and increased atherogenic risk calculated with the ratio of TG to HDL-c and total cholesterol to HDL-c.

Recent prospective studies demonstrate that hyperuricemia is a predictor of IR (5). It was observed that, for every increase of 1 mg/dL in SUAC, there would be a 91% increase in risk of IR. However, the pathophysiological mechanism of the association between hyperuricemia and hyperinsulinemia/IR is not yet clearly established. A double correlation has been proposed; in general, IR and hyperinsulinemia are thought to increase SUAC by reducing renal excretion and increasing production through the hexosemonophosphate shunt (1). Another possible link between hyperuricemia and IR could be hyperuricemia-mediated endothelial dysfunction which may lead to lower insulin uptake by reduced blood flow in peripheral tissues and may worsen the IR (35). Consistent with these pathogenic findings, hyperuricemic patients in our study had significantly higher insulin levels at 0, 30 and 60 minutes. SUAC showed a significantly positive correlation with insulin levels both at fasting and at all estimations following oral glucose loading. We also found that SUA is significantly associated with the HOMA-IR. Cardoso et al. (9) showed the association between MetS and SUAC by IR and they reported that while glycemia was not different, HOMA-IR significantly varied among quartiles of SUAC. In our study, we found a significant correlation between UA and glucose levels only at post challenge 60 minutes. Similarly Ricotti et al. (36) showed that hyperuricemic patients were at increased risk of having a 1-hour post-OGTT glycemia which was also associated with increased metabolic risk.

The association of higher SUAC with higher BP has been reported in adults and children in a number of studies (26,37). The lack of an association between SUA and BP in our sample may be related to the fact that duration of exposure to increased SUAC and related inflammation and oxidative stress was not evaluated in our study. In adults, in addition to microalbuminuria, hyperuricemia is a well-established risk factor for chronic kidney disease (26). However, data concerning the relationship between hyperuricemia and renal injury in OB children are still lacking. We did not find a significant association in our study group. Long-term prospective studies are needed on this subject.

### Study Limitations

Our study has some limitations. We used percentages of the UA according to age and sex but SUAC may be affected by pubertal stage. We did not encounter any UA reference values which took into account sex and pubertal stages in the literature. Comprehensive studies are needed on this issue. The major limitation of our study is the relatively small size of the sample.

## Conclusion

In conclusion, we believe that SUAC is a good alternative to assess cardiometabolic risk, even at a young age. Chronic hyperuricemia appears to be involved in the pathogenesis of metabolic impairment leading to MetS and subsequent comorbidities. The prevention of SUA elevation at an early age by  SUA lowering agents may have a potential protective effect on hyperglycemia, hyperinsulinemia, IR, dislipidemia and hypertension (38). It is feasable to include assessment of UA in routine tests in primary care since its estimation is widely available, very cheap and reliable. We therefore suggest that measurement of SUAC should be included in the assessment protocols of OB/OW children and adolescents. We would also like to add that there is a need for prospective clinical studies to evaluate the clinical significance and to assess the cost effectiveness of measuring routinely SUAC in childhood obesity.

## Figures and Tables

**Table 1:**
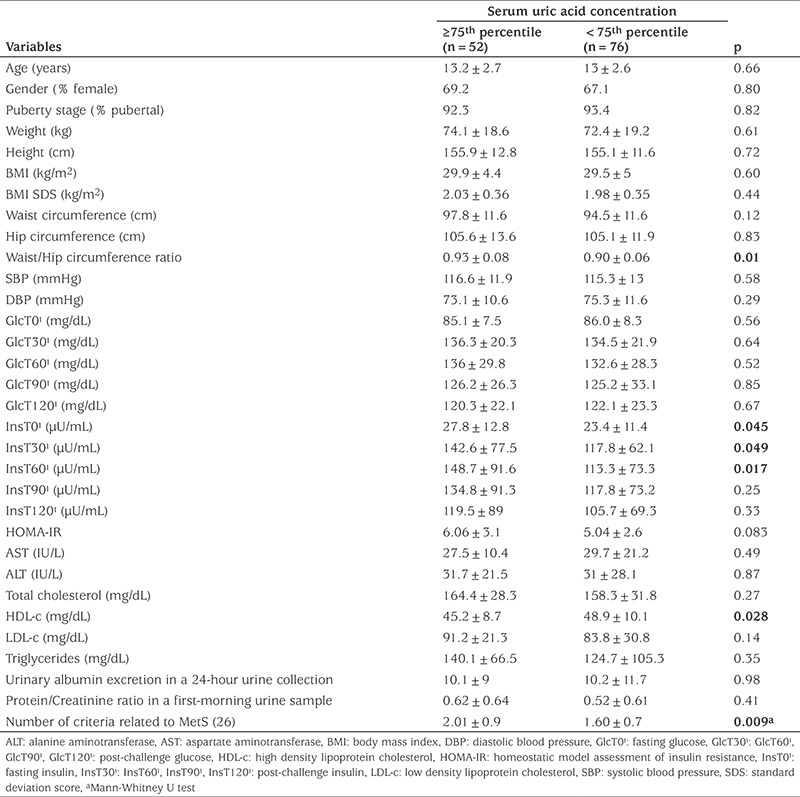
Clinical and laboratory characteristics of the study groups

**Table 2:**
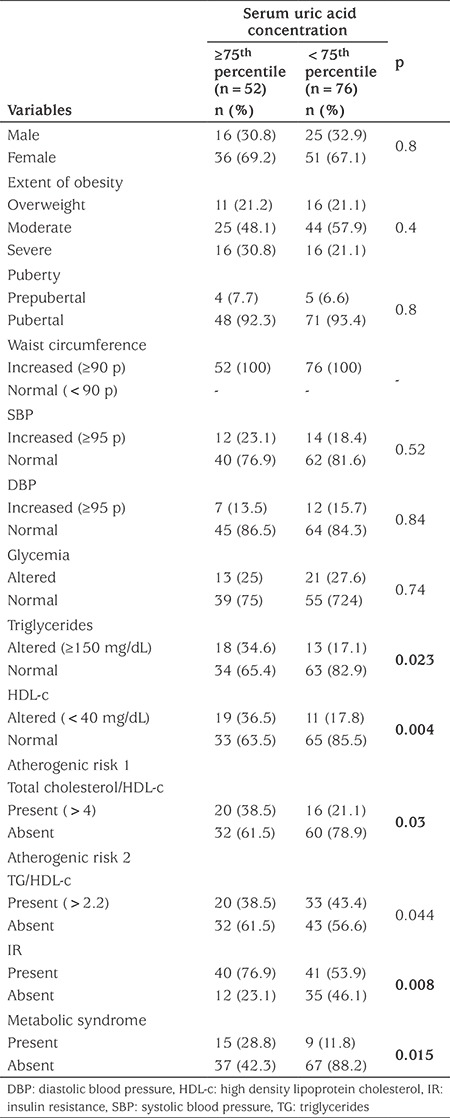
Anthropometric, clinical and metabolic variables of the study population according to their uric acid levels

**Table 3:**
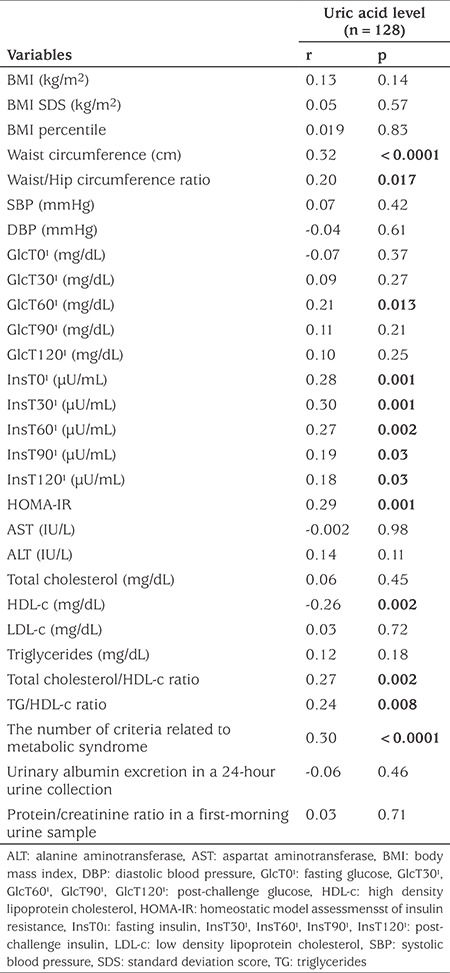
Correlations between serum uric acid levels and risk factors for metabolic syndrome, cardiovascular risk and renal injury
